# Accurate Core-Level Ionization Energies from an Affordable Second-Order Approach

**DOI:** 10.1021/acs.jctc.6c00015

**Published:** 2026-03-18

**Authors:** Dávid Mester, Mihály Kállay

**Affiliations:** † Department of Physical Chemistry and Materials Science, Faculty of Chemical Technology and Biotechnology, 415618 Budapest University of Technology and Economics, Műegyetem rkp. 3., H-1111 Budapest, Hungary; ‡ HUN-REN-BME Quantum Chemistry Research Group, Műegyetem rkp. 3., H-1111 Budapest, Hungary; § MTA-BME Lendület Quantum Chemistry Research Group, Műegyetem rkp. 3., H-1111 Budapest, Hungary

## Abstract

An approach is proposed for the accurate calculation of core-level ionization potentials (IPs) using second-order methods. The assessed theoretical frameworks are based on the iterative second-order algebraic-diagrammatic construction [ADC(2)] and configuration interaction singles with perturbative second-order correction [CIS­(D)] methods. Here, our efficient implementations of IP-ADC(2) and IP-CIS­(D) [ MesterD., ; KállayM., J. Chem. Theory Comput. 2023, 19, 3982–3995 37326360 10.1021/acs.jctc.3c00363PMC10339736] are combined with the core–valence separation (CVS) approximation, thereby enabling ionization from the core region. The approaches exhibit highly favorable scaling behavior: the computational cost is practically cubic, with only a mild prefactor that depends on the number of active core orbitals. Furthermore, the resulting wave function-based methods are combined with spin-scaling techniques and successfully extended to double-hybrid (DH) functionals without any necessary modifications in the implementation of the second-order corrections. The performance of the proposed methods was thoroughly assessed in benchmark calculations. Our results demonstrate that the iterative treatment of double excitations is essential, underscoring the necessity of the more advanced DH ansatz. Moreover, the SOS0-PBE0-2/CVS-IP-ADC(2) approach is highly competitive with more expensive higher-level coupled-cluster methods. Finally, the second-order correction introduces only a negligible overhead in the overall computational time—only about 1 min for a 61-atom azafullerene molecule using triple-ζ basis sets—thereby enabling accurate calculations for extended molecular systems.

## Graphical abstract

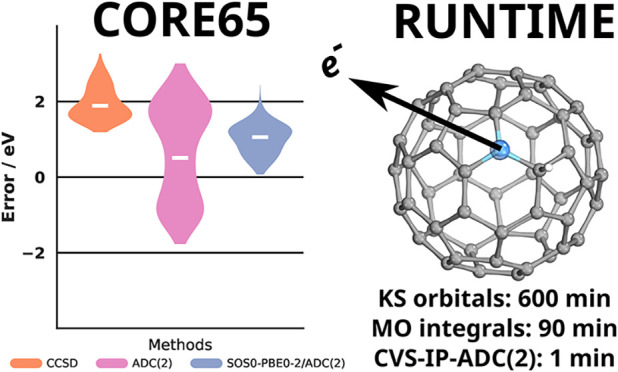

## 1 Introduction

In recent years, significant advances in experimental instrumentation—particularly the development of synchrotron^1^ and free-electron laser radiation sources^2^—have greatly expanded the scope of modern spectroscopy. Building on these developments, X-ray photoelectron spectroscopy (XPS) has emerged as a cornerstone technique in materials science and chemistry.^3–7^ Its strength lies in its ability to provide distinct chemical fingerprints and detailed insight into the molecular and electronic structures of compounds, owing to the localized and element-specific nature of core orbitals. One of the advantages of XPS is that the ionization process is not subject to optical selection rules, making the technique sensitive to both optically bright and optically dark core-excited states. Despite these achievements, developing accurate and efficient theoretical methods to describe such ionized states remains an ongoing and significant challenge in quantum chemistry.

Electronic structure methods can be broadly classified into two categories: wave function-based and density functional theory (DFT)-based approaches. The main advantage of the former is their systematic improvability as higher-level methods within the same formalism provide increasingly accurate results. Their major drawback is the steep computational scaling with system size. In contrast, DFT-based approaches offer much lower computational cost but often suffer from questionable accuracy and lack a straightforward route for systematic improvement. Combined schemes that merge the two frameworks have gained considerable attention in recent years;^8–10^ however, their application to the calculation of core binding energies remains limited due to the absence of appropriate theoretical formalisms and implementations.

Within the family of wave function-based methods, the algebraic-diagrammatic construction (ADC)^11^ and coupled-cluster (CC)^12^ frameworks represent the most reliable and extensively used techniques. The ADC approach originates from the diagrammatic perturbation expansion of the polarization propagator and the Møller–Plesset (MP) partitioning of the Hamiltonian, whereas CC theory relies on the exponential parametrization of the wave function. Both formalisms allow direct computation of ionization potentials (IPs) and indirect inclusion of orbital relaxation effects through coupling to higher excited configurations. In practice, this means that orbital relaxation is not described by explicit orbital optimization but arises from the coupling of the primary one-hole (1h) configurations to higher excited configurations, such as two-hole–one-particle (2h1p) states. The equation-of-motion CC (EOM-CC)^13–15^ framework is based on a similarity-transformed Hamiltonian, which effective operator is diagonalized to obtain final-state wave functions and energies. Starting from an *n*-electron reference, ionization processes are described by diagonalizing the corresponding operator in a determinant basis containing *n* – 1 electrons.^16–20^ Within the ADC family, non-Dyson schemes are most commonly employed, enabling independent calculations of electron-detached and electron-attached states.^21–25^ Continued methodological developments have also yielded several efficient electron propagator methods for accurate ionization potential calculations.^26–29^

The eigenvalue equations are typically solved using iterative diagonalization schemes that produce the lowest eigenvalues.^30,31^ Because core-level transitions occur in the high-energy X-ray region, such calculations for large systems become computationally demanding when performed using standard procedures. The core–valence separation (CVS)^32^ approximation addresses this issue by neglecting couplings between core- and valence-excited configurations. This approximation exploits the large energetic and spatial separation between core and valence orbitals, allowing the Hamiltonian to be block-diagonalized and the equations to be solved within the targeted core-excited subspace. The CVS approximation was first introduced within the ADC framework^32,33^ and later extended to EOM-CC theory.^34^ Several interpretations have been proposed; the two most straightforward brute-force variants either set the corresponding molecular integrals to zero^32^ or project out the relevant matrix elements after their evaluation.^34^ These approaches are straightforward to implement in existing codes with minimal modification but can involve unnecessary computational effort. However, significant progresses have been made toward more efficient implementations.^35–37^ The CVS-EOM-CC and CVS-ADC methods have become increasingly prominent, largely due to the pioneering work of Coriani,^35,38–40^ Matthews,^41–44^ Dreuw,^45–48^ and their co-workers.

Core-level ionization energies can be computed by combining the IP formalism with the CVS approximation. In recent years, several accurate CVS-EOMIP-CC^41,42,49^ and CVS-IP-ADC^50–55^ methods have been proposed, enabling accurate calculation of core binding energies. The excellent performance of these approaches has motivated further development, particularly toward reducing computational costs.^56–58^ Another active area of research concerns the treatment of systems with strong multireference character. To address this challenge, Evangelista and co-workers proposed the generalized active space-driven similarity renormalization group formalism, which provides a promising framework for describing such systems.^59–64^

Excitations from the core region can also be computed using DFT methods. The most straightforward approach combines the time-dependent (TD) formalism with the CVS approximation;^65,66^ however, several more advanced schemes have also been proposed.^67–70^ The accuracy of CVS-TDDFT can be substantially improved by employing tailored exchange–correlation (XC) functionals^71–74^ or by combining it with second-order wave function-based approaches, thereby reaching the double-hybrid (DH) level.^75^ The computation of ionizations is less straightforward. The first ionization energy can, in the simplest sense, be interpreted according to Koopmans’ theorem as equal to the negative of the Kohn–Sham (KS) orbital energy of the electron being removed.^76,77^ DFT methods that directly compute higher-order corrections to this quantity in a single step are still rather rare in practice, although there are promising attempts in this direction.^78–80^ Alternatively, appealing approaches for computing excitations and ionization energies are the orbital-optimized methods, where the orbitals are variationally relaxed for each state individually.^81–86^ It is worth noting that this strategy is also gaining traction within wave function-based methods.^87–91^ Despite their excellent numerical performance, these approaches face several technical challenges in most cases. For instance, the non-Aufbau determinant can easily lead to variational collapse, the resulting final states are not orthogonal to the ground state, and the calculations often require substantial user intervention. Finally, we highlight the Slater transition concept as a noteworthy approach for obtaining accurate core binding energies within the DFT framework, which was recently benchmarked by Herbert and co-worker.^92^

In this paper, we propose a cost-effective approach for calculating accurate core binding energies. First, we extend the second-order wave function-based methods underlying our frameworks to the computation of ionizations from the core region, employing the IP formalism in conjunction with the CVS approximation. We then briefly discuss possible spin-scaling techniques and the extension of these methods to the DH-TDDFT level. The performance of the resulting approaches is carefully evaluated using widely adopted benchmark sets from the literature, and their favorable computational efficiency is demonstrated through a representative example.

## 2 Theory

### 2.1 Second-Order Ionization Potentials

The extension of second-order methods, such as configuration interaction singles with perturbative doubles [CIS­(D)][Bibr ref93] and second-order ADC [ADC(2)],^94,95^ to the calculation of ionization energies has been discussed in earlier studies.^21–24,80,96^ For the computation of core binding energies, a brief outline of the underlying theoretical framework is required; however, to preserve the compactness of this manuscript, only a short summary is provided here.

We begin by introducing the theory based on the perturbative CIS­(D) method. Its starting point is the formal extension of the CIS approach^97^ to ionization potentials, using a Hartree–Fock (HF) reference Φ_0_. Defining a generic ionization operator *Ĉ*_1_ = ∑_*i*_ *c*_*i*_ *i* ^–^, where *i* ^–^ is an annihilation operator acting on occupied spatial orbital *i* with *c*_*i*_ as the corresponding coefficient, the ionized state is expanded as a linear combination of singly ionized determinants Φ_*i*_: *Ĉ*_1_Φ_0_ = ∑_*i*_ *c*_i_Φ_*i*_. Projection onto the subspace spanned by ionized determinants yields the formal IP-CIS eigenvalue equations

1 $$\left\langle \Phi_{k}\right|Ĥ\left|{Ĉ}_{1}\Phi_{0}\right\rangle =-\varepsilon_{k}c_{k}$$

Here, the eigenvalues corresponding to the negative occupied orbital energies interpreted as ionization energies, ω_*k*_^IP‑CIS^ = −ε_*k*_, while the eigenvectors form an orthonormal set of unit vectors, from which the IP-CIS eigenstate follows: Ψ_*k*_^IP‑CIS^ = *Ĉ*_1_Φ_0_ = ∑_*i*_δ_*ik*_Φ_*i*_ = Φ_*k*_.

The perturbative second-order (D) correction associated with ionization from the *k*th orbital can be interpreted as a CIS­(D) calculation carried out on the corresponding ionized determinant

2 $$E_{k}^{\left(D\right)}=\left\langle \Phi_{k}\right|\mathrm{V̂}\left|{Ĉ}_{2}\Phi_{0}\right\rangle +\left\langle \Phi_{k}\right|\mathrm{V̂}\left|{\mathrm{T̂}}_{2}{Ĉ}_{1}\Phi_{0}\right\rangle$$

In this equation, the double excitation coefficients and ground-state first-order amplitudes appear in the operators *Ĉ*_2_ = ∑_*ija*_ *c*_*ia*,*j*_ *a* ^+^ *i* ^–^ *j* ^–^ and ${\mathrm{T̂}}_{2}=\frac{1}{2}\sum_{\mathrm{ijab}}t_{\mathrm{ia},\mathrm{jb}}a^{+}i^{-}b^{+}j^{-}$ , where *a* ^+^ denotes a creation operator acting on virtual spatial orbital *a*. Within the first term on the right-hand side, the double excitation coefficients are obtained from the perturbative expression

3 $${Ĉ}_{2}\Phi_{0}=\sum_{\mathrm{ija}}c_{\mathrm{ia},j}\Phi_{\mathrm{ij}}^{a}=\sum_{\mathrm{ija}}\frac{\left\langle \Phi_{\mathrm{ij}}^{a}\right|\mathrm{V̂}\left|{Ĉ}_{1}\Phi_{0}\right\rangle }{\varepsilon_{i}+\varepsilon_{j}-\varepsilon_{a}+\omega_{k}^{\mathrm{IP}‐\mathrm{CIS}}}\Phi_{\mathrm{ij}}^{a}=\sum_{\mathrm{ija}}\frac{V_{\mathrm{ia},j}^{k}}{D_{\mathrm{ij}}^{a}+\omega_{k}^{\mathrm{IP}‐\mathrm{CIS}}}\Phi_{\mathrm{ij}}^{a}=\sum_{\mathrm{ija}}\frac{-\left(\mathrm{ia}|\mathrm{jk}\right)}{D_{\mathrm{ij}}^{a}+\omega_{k}^{\mathrm{IP}‐\mathrm{CIS}}}\Phi_{\mathrm{ij}}^{a}$$

while the second term can be separated into disconnected and connected contributions. Since the disconnected part, using

4 $${\mathrm{T̂}}_{2}\Phi_{0}=\frac{1}{2}\sum_{\mathrm{ijab}}t_{\mathrm{ia},\mathrm{jb}}\Phi_{\mathrm{ij}}^{\mathrm{ab}}=\frac{1}{2}\sum_{\mathrm{ijab}}\frac{\left(\mathrm{ia}|\mathrm{jb}\right)}{D_{\mathrm{ij}}^{\mathrm{ab}}}\Phi_{\mathrm{ij}}^{\mathrm{ab}}$$

reproduces the conventional second-order MP (MP2) energy, the perturbative correction to the ionization energy becomes^80^

5 $$\omega_{k}^{\left(D\right)}=E_{k}^{\left(D\right)}-E^{\mathrm{MP}2}=\sum_{\mathrm{ija}}V_{\mathrm{ia},j}^{k}\left(2c_{\mathrm{ia},j}-c_{\mathrm{ja},i}\right)-\sum_{\mathrm{jab}}\left(\mathrm{ka}|\mathrm{jb}\right)\left(2t_{\mathrm{ka},\mathrm{jb}}-t_{\mathrm{ja},\mathrm{kb}}\right)=\sum_{\mathrm{ija}}V_{\mathrm{ia},j}^{k}{\mathrm{c̃}}_{\mathrm{ia},j}-\sum_{\mathrm{jab}}\left(\mathrm{ka}|\mathrm{jb}\right){\mathrm{t̃}}_{\mathrm{ka},\mathrm{jb}}$$

Thus, the final ionization energy is obtained as ω^IP‑CIS(D)^ = ω^IP‑CIS^ + ω^(D)^. We note that these expressions recover the second-order self-energies in the diagonal and frequency-independent approximations, corresponding to the so-called ΔMP2 approach.^98–101^

For the IP-ADC(2) method, in practice, a nonlinear eigenvalue equation is solved iteratively

6 $${Ã}^{\mathrm{IP}‐\mathrm{ADC}\left(2\right)}\left(\omega^{\mathrm{IP}‐\mathrm{ADC}\left(2\right)}\right)c=\omega^{\mathrm{IP}‐\mathrm{ADC}\left(2\right)}c$$

where **Ã** ^IP‑ADC(2)^ denotes the so-called effective ADC(2) Jacobian, and ω^IP‑ADC(2)^ is the corresponding ionization energy. The eigenvalue problem is typically solved using the conventional Davidson algorithm,^30,31^ whose rate-determining step is the evaluation of the matrix–vector product **σ** = **Ã** ^IP‑ADC(2)^ **c**. Within the non-Dyson IP-ADC(2) scheme, the contributions are explicitly given by^80,96^

7 $$\sigma_{i}^{\mathrm{IP}‐\mathrm{ADC}\left(2\right)}=-\varepsilon_{i}c_{i}-\sum_{\mathrm{jka}}\left(\mathrm{ik}|\mathrm{ja}\right){\mathrm{c̃}}_{\mathrm{ja},k}-\frac{1}{2}\sum_{\mathrm{jkab}}\left(\mathrm{ia}|\mathrm{kb}\right){\mathrm{t̃}}_{\mathrm{ja},\mathrm{kb}}c_{j}-\frac{1}{2}\sum_{\mathrm{jkab}}\left(\mathrm{ja}|\mathrm{kb}\right){\mathrm{t̃}}_{\mathrm{ia},\mathrm{kb}}c_{j}$$

During the iterative procedure, contractions very similar to those required for IP-CIS­(D) calculations have to be performed. However, since the eigenvectors in ADC(2) are not unit vectors, the final state is expanded correctly, at least within the singles space, as Φ_*n*–1_ = ∑_*i*_ *c*_*i*_Φ_*i*_ ≠ ∑_*i*_δ_*ij*_Φ_*i*_. Consequently, the intermediate **V** is given by *V*_*ia*,*j*_ = −∑_*k*_(*ia*|*jk*)*c*_*k*_, and the double excitation coefficients are evaluated using the ionization energies obtained in the current iteration (see eq 3). We note that independent efficient implementations of IP-ADC(2) were also reported by Sokolov and Banerjee.^24^

### 2.2 Second-Order Core Binding Energies

Core binding energies correspond to ionizations from deeply bound core orbitals, lying in the high-energy X-ray domain of the spectrum. The Davidson procedure described above yields the energetically lowest IP-ADC(2) eigenvalues of eq 6. Consequently, computing core binding energies using standard approaches would be highly inefficient at this level. By invoking the CVS approximation,^32^ couplings between core and valence excitations can be neglected *a priori*, allowing the equations to be solved exclusively within the target subspace. From this point on, we distinguish between active (*I*) and inactive (*i*) occupied orbitals for clarity. The active orbitals typically correspond to the set of core orbitals from which the ionization occurs, while the inactive orbitals comprise all other correlated occupied orbitals. In the derived working equations, all contributions that vanish as a consequence of the integral-based separation^32^ are omitted from the outset, and only those terms that provide a non-negligible contribution to the ionization process are retained.

Applying the CVS approximation to the expressions in the previous section is relatively straightforward. Before presenting the working equations, we briefly discuss the density fitting (DF) approximation, in which the matrix **K** with elements *K*_*ia*,*jb*_ = (*ia*|*jb*) is factorized as **K** = **IV** ^–1/2^ **V** ^–1/2^ **I** ^T^ = **JJ** ^T^, with

8 $$J_{\mathrm{ia}}^{P}=\sum_{Q}I_{\mathrm{ia}}^{Q}V_{\mathrm{PQ}}^{-1/2}$$

Here, *P* and *Q* label elements of the DF auxiliary basis, while *I*_*ia*_^*Q*^ and *V*_*PQ*_ denote the three- and two-center Coulomb integrals, respectively. This factorization avoids the expensive storage and transformation of four-center two-electron integrals. Employing both the DF and CVS approximations, the perturbative (D) correction associated with ionization from orbital *K* becomes

9 $$\omega_{K}^{\mathrm{CVS}‐\left(D\right)}=\sum_{\mathrm{Ija}}V_{I,\mathrm{ja}}^{K}\left(2c_{I,\mathrm{ja}}-c_{\mathrm{Ia},j}\right)+\sum_{\mathrm{Ija}}V_{\mathrm{Ia},j}^{K}\left(2c_{\mathrm{Ia},j}-c_{I,\mathrm{ja}}\right)$$

where *V*_*I*,*ja*_^*K*^ = −∑_*Q*_ *J*_*IK*_^*Q*^ *J*_*ja*_^*Q*^ and *V*_*Ia*,*j*_^*V*^ = −∑_*Q*_ *J*_*Ia*_^*Q*^ *J*_*jK*_^*Q*^. Due to the CVS and frozen-core approximations, contributions from ground-state doubles amplitudes vanish, and, because the summation over occupied orbital indices is restricted to active and inactive subsets, two distinct sets of **V** intermediates and double excitation coefficients must be introduced. The computational cost of evaluating CVS-IP-CIS­(D) ionization energies is particularly favorable. The rate-determining step is the computation of the **V** intermediates, which scales as *N*_act_ *N*_inact_ *N*_virt_ *N*_aux_, where *N*_act_ and *N*_inact_ denote the numbers of active and inactive occupied orbitals, respectively, *N*_virt_ is the number of virtual orbitals, and *N*_aux_ is the number of auxiliary functions. Since *N*_act_ is typically very small, the effective scaling is practically cubic with a small prefactor. We note that while the frozen-core approximation follows naturally from the CVS treatment,^32^ its use may limit the ultimate accuracy of systematically convergent methods, such as EOM-CC and ADC. However, in the present context, the explicit computation of MP2 amplitudes would be particularly inefficient since the computational cost of the CVS-IP problem scales only cubically with a mild prefactor, and the adoption of the frozen-core approximation is therefore primarily motivated by computational efficiency, which defines the intended scope of this work. Accordingly, as shown later by wall-clock timings (see Section 4.4), the rate-determining step in these calculations is the determination of the HF reference orbital set itself. Furthermore, the problematic virtual–virtual part of the three-center integral list is not required here—unlike in CIS­(D)—and thus the memory requirement is reduced to storing the *J*_*ia*_^*P*^ array. The computation and storage of four-index arrays are entirely avoided within this formalism, and none of the required arrays contains more than one virtual index.

Analogously, the DF and CVS approximations can be applied to IP-ADC(2) to obtain core binding energies. The elements of the **σ** vector in the resulting CVS-IP-ADC(2) method are given as

10 $$\sigma_{I}^{\mathrm{CVS}‐\mathrm{IP}‐\mathrm{ADC}\left(2\right)}=-\varepsilon_{I}c_{I}-\sum_{\mathrm{KQ}}J_{\mathrm{IK}}^{Q}\sum_{\mathrm{ja}}J_{\mathrm{ja}}^{Q}\left(2c_{K,\mathrm{ja}}-c_{\mathrm{Ka},j}\right)-\sum_{\mathrm{kQ}}J_{\mathrm{Ik}}^{Q}\sum_{\mathrm{Ja}}J_{\mathrm{Ja}}^{Q}\left(2c_{\mathrm{Ja},k}-c_{J,\mathrm{ka}}\right)$$

where the **V** intermediates required for computing the double excitation coefficients are given as follows: *V*_*I*,*jb*_ = −∑_*KQ*_ *J*_*KI*_^*Q*^ *c*_*K*_ *J*_*jb*_^*Q*^ and *V*_*Ib*,*j*_ = −∑_*KQ*_ *J*_*Ib*_^*Q*^ *J*_*Kj*_^*Q*^ *c*_*K*_. The memory requirements and scaling remain similar to those of CVS-IP-CIS­(D), although the procedure is iterative. Nevertheless, this does not pose practical difficulties as computing ionization energies is still orders of magnitude cheaper than obtaining the reference orbitals.

The methods presented here can be freely combined with spin-scaling techniques. In such cases, the same- and opposite-spin components of the second-order terms are scaled independently.^102,103^ Recently, Tajti and co-workers^104^ demonstrated that applying the spin-opposite-scaling (SOS) approach significantly improves the accuracy of valence ionization energies at the IP-ADC(2) level. We examine this scheme in more detail later. Since several interpretations of spin scaling for excited-state calculations exist in the literature,^105–107^ we emphasize that our implementation follows the suggestion of Hättig and co-workers.^107,108^ Within this framework, for CVS-IP-SOS-CIS­(D) and CVS-IP-SOS-ADC(2), a single scaling factor of 1.3 is applied to all opposite-spin contributions, while same-spin contributions are neglected.

The methods described above can also be extended to the calculation of core binding energies within the TDDFT formalism for DH functionals, employing a CIS­(D)^109^- or ADC(2)-based^110^ ansatz. The anticipated advantages of this approach originate from a more balanced description of the reference electronic structure provided by KS orbitals combined with scaled second-order correlation contributions. In particular, KS orbital energies are typically much closer to experimental ionization energies than HF orbital energies, while the empirically scaled second-order correction provides a more balanced improvement of the results. In our previous work, we extended the DH-TDDFT approach to the computation of ionization potentials.^80^ A noteworthy aspect of the resulting expressions is that XC DFT contributions persist solely during the construction of the KS reference orbitals and vanish entirely when solving eq 1. This implies that even in the hybrid TDDFT regime, the ionization energy remains equal to the negative occupied orbital energy. Moreover, the implementation is simplified by the fact that higher-order derivatives of the XC energy are not needed. These results can be improved by adding second-order corrections—either perturbatively or iteratively—corresponding to the IP-CIS­(D)- or IP-ADC(2)-based DH analogues.^80^ To compute core binding energies, the CVS approximation must be applied to these expressions. As discussed above, this means that only the contributions from eqs 9 or 10 are evaluated according to the specified ansatz, and these contributions are scaled with the correlation mixing factor associated with the chosen DH functional.^80,110^

## 3 Computational Details

All calculations were carried out using the development version of the Mrcc suite of quantum chemical programs.^111,112^ In this study, the correlation-consistent core–valence *X*-tuple-ζ basis sets recontracted for exact two-component (X2C) relativistic calculations, cc-pCV*X*Z-X2C (X = T and Q),^113–115^ were employed as atomic orbital basis sets. The DF approximation was applied at both the HF/KS and post-HF/KS levels. The corresponding auxiliary basis sets were generated using the AutoAUX algorithm.^116^ For the DFT contributions, the built-in XC functional of Perdew, Burke, and Ernzerhof (PBE)^117^ was used. This choice will be justified later. The default adaptive integration grid of the Mrcc package was employed. The frozen-core approximation was employed in all post-HF/KS calculations. To account for scalar relativistic effects, the spin-free X2C one-electron (SFX2C-1e) Hamiltonian was utilized,^118,119^ which was implemented in Mrcc for this study. The reported computation times correspond to wall-clock times measured on an Intel Xeon E5-2609 v4 @ 1.70 GHz processor using 8 cores.

For the benchmark calculations, two data sets reported in the literature were used. In the first set, the core binding energies of small molecules containing one or two first-row elements were evaluated. Molecular geometries were taken from the study of Cheng and co-workers,^49^ which correspond to experimental structures for diatomic molecules and high-level CC optimized geometries for larger systems. This set was intended to be supplemented with molecules from the data set employed by Head-Gordon and co-workers.^91^ Since the two benchmark selections overlap significantly, we additionally included only the H_2_CO molecule from the latter. The resulting set contains 14 molecules and 23 ionizations. Experimental values were used as reference data, and the performance of our methods was compared to higher-level CC approaches, such as the CVS-EOMIP-CCSD^18,49,120^ and CVS-EOMIP-CCSDT methods,^18,49,121,122^ which also include scalar relativistic contributions using the SFX2C-1e scheme. These values were taken from ref 49. Since this study does not include the H_2_CO molecule, the statistical analysis for the CC methods was therefore performed for only 21 ionizations. All calculations were performed using the cc-pCVQZ-X2C basis sets.

In the second set, the performance of our methods for relatively large organic systems was assessed using the CORE65 benchmark set,^123^ which contains 65 core binding energies for 32 molecules with up to 14 atoms. This benchmark set encompasses a wide range of chemical environments, bonding types, and the most common functional groups. The molecular geometries were taken from the original publication,^123^ where they were optimized at the DFT level. Experimental data were used as reference values for this set as well, while our results were compared to those obtained with IP-CVS-EOM-CCSD and its more advanced version including perturbative triple substitutions (IP-CVS-EOM-CCSD*).^18,58,124^ These values, which also include scalar relativistic contributions using the SFX2C-1e scheme, were taken from ref 58. All calculations were carried out using the cc-pCVTZ-X2C basis sets. In all cases, K-edge ionization potentials were computed for the C, N, O, and F atoms. For the first set, the numbers of core binding energies were 9, 5, 7, and 2, respectively, while for the second set they were 30, 11, 21, and 3, respectively. For convenience, the CVS-IP prefix will be omitted from the names of the approaches in the text and figures hereinafter. In some cases, however, such as in the figure captions, it is retained to ensure an unambiguous reference to the methods used in other publications.

## 4 Results and Discussion

### 4.1 Small-Molecule Compilation

Before discussing the results, we would like to justify our choice of the DH functional. In this work, the performance is discussed in detail only for the SOS0-PBE0-2 functional;^125^ however, in this case, we examine the performance of both the CIS­(D)- and ADC(2)-based ansätze. This choice was made to maintain the compactness of the manuscript, but, in summary, we can conclude that this functional provided the highest accuracy. Calculations were also performed using several of the most popular functionals available in the literature, such as B2PLYP,^9^ B2GPPLYP,^126^ DSD-PBEP86,^127^ PBE0-2,^128^ PBE-QIDH,^129^ and SOS0-PBE-QIDH.^125^ For most of these functionals, we were not able to improve upon the results obtained with the purely wave function-based ADC(2) and CIS­(D) counterparts. This finding is consistent with our previous results, which led to similar conclusions when investigating core excitation energies.^75^ In that case as well, the SOS0-PBE0-2 functional provided the best results, while the performance of most other functionals was not convincing. Accordingly, in this study, we discuss only the results obtained with this functional; however, all raw numerical data for the other functionals are provided in the Supporting Information (SI). In addition, the corresponding statistical measures are also available, including the mean error (ME), mean absolute error (MAE), standard deviation (SD) of the errors, root-mean-square error, maximum absolute error (MAX), and the span of the errors.

We first discuss the results for the small-molecule benchmark set. The violin plots summarizing the error measures are presented in Figure 1. From the shapes of the plots, several important observations can be made; however, for better comparability, we also report exact numerical values. It is not surprising that the full CCSDT method performs exceptionally well in every respect. The most favorable metrics were obtained for this approach, with ME and MAE values of 0.06 and 0.14 eV, respectively, accompanied by an exceptionally low SD of 0.17 eV. The MAX error is remarkably small, only 0.54 eV, while the overall error span is 0.70 eV. These results clearly demonstrate the outstanding balance and reliability of the method. For CCSD, the distribution is noticeably more elongated, and a significant blueshift can also be observed. The former corresponds to an increase in SD and span, reaching 0.41 and 1.41 eV, respectively, which are still highly acceptable. The pronounced shift, however, indicates a substantial overestimation of ionization energies, which occurs in a relatively consistent manner. Since all values are overestimated, the ME and MAE are identical, both being 1.82 eV, with a MAX of 2.54 eV.

**Figure 1:**
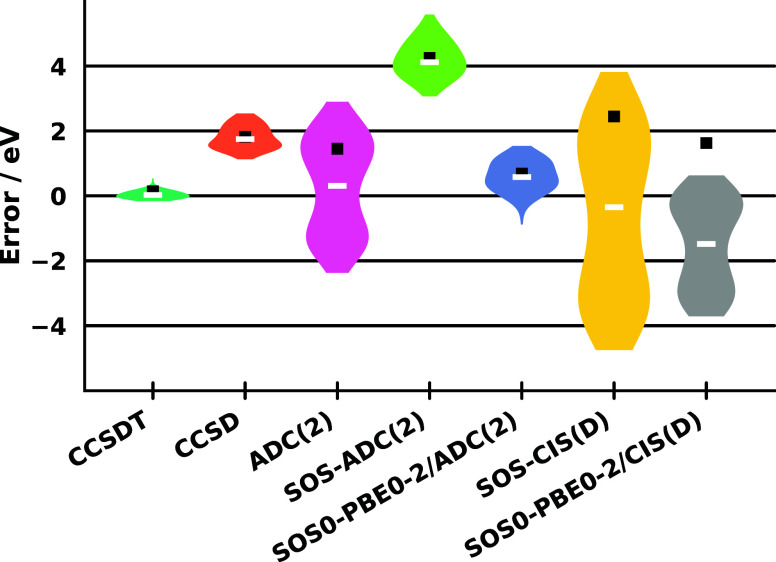
Error measures for the core binding energies of the small-molecule benchmark set using the cc-pCVQZ-X2C basis sets. The ME and MAE are indicated by a white line and a black square, respectively. The CVS-EOMIP-CCSD and CVS-EOMIP-CCSDT values were taken from ref 49. All values include SFX2C-1e relativistic contributions.

In comparison, for ADC(2), the distribution becomes even more elongated. In this case, the SD and span are considerable, namely 1.58 and 5.27 eV, respectively. However, as can also be seen from the plot, the positive and negative errors largely cancel each other out, resulting in an impressive ME. The MAE is also somewhat improved, reaching 1.45 eV, compared to CCSD. Based on these observations, we can conclude that the method is generally accurate but lacks precision. For the SOS-ADC(2) approach, both SD and span decrease significantly, indicating higher precision. Unfortunately, a notable blueshift appears simultaneously, meaning that the core binding energies are significantly, though fairly consistently, overestimated. Thus, the iterative second-order methods exhibit complementary advantages: ADC(2) offers excellent ME and MAE values, while SOS-ADC(2) provides lower SD and span. Nevertheless, in their current form, neither approach can compete with CCSD in overall performance.

A similar trend can be observed for the perturbative CIS­(D) method, although the errors are substantially larger. The CIS­(D) results are not displayed in the figure for clarity. Nevertheless, it can be stated that the ionization energies are seriously underestimated, accompanied by very large SD and span values. The SOS-CIS­(D) variant shows a notable improvement, although considerable errors remain. The ME is highly acceptable at −0.58 eV, but it is accompanied by an SD of approximately 2.7 eV and a span approaching 8.5 eV. As will be shown later, the computational cost difference between ADC(2) and CIS­(D) is negligible (see Section 4.4); therefore, there is no justification for using CIS­(D) and its variants. The results also clearly demonstrate the importance of treating double excitations iteratively.

Now, let us turn our attention to the DH functionals. In general, for both ansätze, a clear improvement in performance can be observed. For the iterative approach, applying the DH functional, compared to ADC(2), significantly reduced the SD and span, while relative to SOS-ADC(2), it effectively corrected the pronounced overestimation of core binding energies. This yielded excellent ME and MAE values of 0.56 and 0.68 eV, respectively, for SOS0-PBE0-2/ADC(2). At the same time, the SD and span remain 0.59 and 2.43 eV. This substantial improvement indicates that we nearly achieved CCSD-level precision, while enhancing the accuracy by more than 1 eV compared to the computationally more demanding method. The improvement is also significant for the perturbative approach; however, for the reasons discussed above, its application is not warranted.

### 4.2 CORE65 Compilation

The performance of the methods was also assessed using the CORE65 benchmark set,^123^ which contains molecules more relevant from an application perspective. The results are depicted in Figure 2. Overall, the observed trends closely follow those discussed above; therefore, only a brief summary is provided here. The highest-level method, CCSD*, which includes triple substitutions, unsurprisingly delivered the best overall performance. Although the errors are somewhat larger than those obtained for the small-molecule set with CCSDT, the results are not directly comparable, and the differences are minor. The ME and MAE are both 0.43 eV, while the SD remains exceptionally low at 0.22 eV. The core binding energies are slightly and consistently overestimated, with a MAX of 1.38 eV and a somewhat smaller span of 1.25 eV. For the CCSD approach, noticeably less favorable metrics were obtained, underscoring the importance of triple substitutions for achieving highly accurate results. The corresponding distribution is more elongated and significantly blue-shifted, with ME and MAE values exceeding 2 eV. The SD remains moderate and acceptable at 0.53 eV, whereas the MAX increases to 3.27 eV.

**Figure 2:**
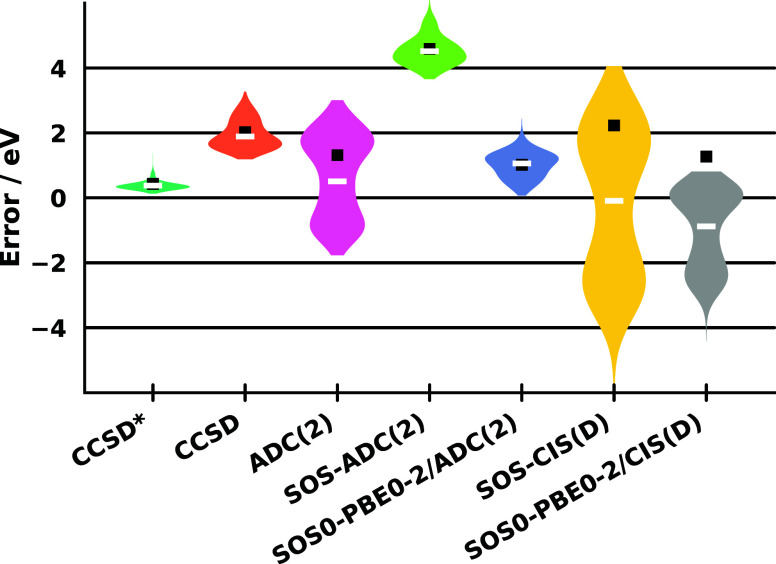
Error measures for the core binding energies of the CORE65 benchmark set using the cc-pCVTZ-X2C basis sets. The ME and MAE are indicated by a white line and a black square, respectively. The IP-CVS-EOM-CCSD and IP-CVS-EOM-CCSD* values were taken from ref 58. All values include SFX2C-1e relativistic contributions.

The ADC(2) and SOS-ADC(2) methods exhibit behavior similar to that observed previously. Compared with CCSD, ADC(2) yields more favorable ME and MAE values; notably, the ME is very close to that obtained with CCSD*. However, the SD and span are less satisfactory. The SOS-ADC(2) approach mitigates this issue to some extent but introduces a pronounced blueshift, leading to increased ME and MAE values. The SOS-CIS­(D) method represents a considerable improvement over CIS­(D); nevertheless, the errors remain significant, and the approach is not competitive with ADC(2) or its variants.

The application of DH functionals continues to substantially improve the results relative to their corresponding wave function-based counterparts. For SOS0-PBE0-2/ADC(2), the method retains the favorable precision of SOS-ADC(2), as indicated by low SD and span values of 0.50 and 2.37 eV, respectively. At the same time, the pronounced overestimation of ionization energies is effectively corrected, resulting in significantly reduced ME, MAE, and MAX values compared with SOS-ADC(2). Specifically, the corresponding metrics—1.02, 1.02, and 2.44 eV—are markedly superior to those obtained with CCSD, while the precision remains practically identical, surpassing it in terms of overall performance.

In practical applications, peak spacing within a spectrum is essential for distinguishing chemically inequivalent atoms. Accordingly, assessing relative ionization energies is essential for evaluating the performance of a given method. To this end, peak separations were analyzed for the CORE65 benchmark set by evaluating ionization energy differences between chemically inequivalent atoms of the same element. Specifically, for molecules containing multiple nonequivalent atomic sites (e.g., the carbonyl and hydroxyl oxygen atoms in CH_3_COOH), the energy differences between the corresponding core ionizations were determined. In each case, the separation was referenced to the lowest ionization energy of the given element. This resulted in a data set of 14 peak separations, based on experimental reference energies, spanning from 0.28 to 4.69 eV. Because these values vary by more than an order of magnitude, relative error metrics were also examined, including the mean relative error $\mathrm{MRE}=\frac{1}{n}\sum_{i}\frac{\omega_{i}-\omega_{i}^{\mathrm{ref}}}{\omega_{i}^{\mathrm{ref}}}$ , mean absolute relative error (MARE), and the SD of the relative error, $\sqrt{\frac{1}{n}\sum_{i}{\left(\frac{\omega_{i}-\omega_{i}^{\mathrm{ref}}}{\omega_{i}^{\mathrm{ref}}}-\mathrm{MRE}\right)}^{2}}$ . The corresponding statistical measures are summarized in Table 1.

**Table 1:** Error Measures for Peak Separations in the CORE65 Benchmark Set, Comparing Absolute (Upper Panel, in eV) and Relative Errors (Lower Panel)^a^

	CCSD*	CCSD	ADC(2)	SOS-ADC(2)	SOS0-PBE0-2/ADC(2)	SOS-CIS(D)	SOS0-PBE0-2/CIS(D)
ME	–0.18	–0.14	0.06	0.07	0.03	0.54	0.31
MAE	0.29	0.31	0.48	0.43	0.34	0.86	0.58
SD of errors	0.37	0.37	0.60	0.56	0.50	1.09	0.85
							
MRE	–0.31	–0.24	–0.16	–0.12	–0.11	0.11	0.04
MARE	0.33	0.29	0.34	0.30	0.25	0.45	0.31
SD of rel. errors	0.44	0.35	0.43	0.39	0.35	0.52	0.41

a Further details on the technical aspects are provided in the main text.

When considering absolute errors, the performance of the methods largely follows previously observed trends. ADC(2)-based approaches exhibit excellent MEs, consistently outperforming both CCSD* and CCSD. However, with respect to the MAE and the SD of the error, higher-level wave function-based methods generally perform better. Nevertheless, the proposed approaches remain highly competitive, particularly SOS0-PBE0-2/ADC(2). A more surprising trend emerges when relative errors are considered. In this comparison, ADC(2)-based methods clearly outperform both CCSD and CCSD*, further highlighting the practical applicability of these affordable approaches.

### 4.3 Performance by Element

The performance of the methods is also important when analyzed for individual chemical elements. Since the exceptional accuracy of the approaches including triple substitutions is well established, and the perturbative second-order approaches are not competitive, we provide a detailed comparison only between the CCSD and SOS0-PBE0-2/ADC(2) methods for the sake of clarity. All molecules used throughout this study were included in the comparison; however, since the two benchmark sets overlap, duplicate entries were removed. The CORE65 benchmark set^123^ is more extensive and was therefore fully retained, with the unique molecules from the small-molecule compilation merged into it. These molecules are HF, F_2_, and N_2_O. As a result, the CORE65 benchmark set was supplemented with two additional F, two N, and one O K-edge ionizations. The results are summarized in Figure 3. The analysis of the plots reveals interesting correlations that could not be observed when considering the benchmark sets as a whole. Specifically, for every element, the SOS0-PBE0-2/ADC(2) method yields more accurate results. Interestingly, as the core binding energy increases, the overestimation by CCSD becomes more pronounced, whereas for SOS0-PBE0-2/ADC(2), it decreases. Accordingly, when comparing the two approaches, the deviation between the MEs and MAEs for carbon is only 0.3 eV, while for nitrogen and oxygen, it increases to 1 and 2 eV, respectively. At the same time, the precision of the two methods is practically identical: for each element, the CCSD approach yields SD values at most 0.1 eV smaller, which is a negligible difference. The SD values for all elements fall within the range of 0.3–0.5 eV. The MAX errors are lower for the DH functional, whereas the span is slightly more favorable for CCSD. Consistent with the above observations, the difference in MAX values increases as the ionization energy increases: it is only 0.3 eV for carbon but rises to 2 eV for fluorine. The differences in the span are milder—about 0.2 eV for carbon, approximately 0.3 eV for oxygen and fluorine, and 0.6 eV for nitrogen. From this comparison, it can be clearly concluded that the SOS0-PBE0-2/ADC(2) approach is highly competitive with the considerably more expensive CCSD method.

**Figure 3:**
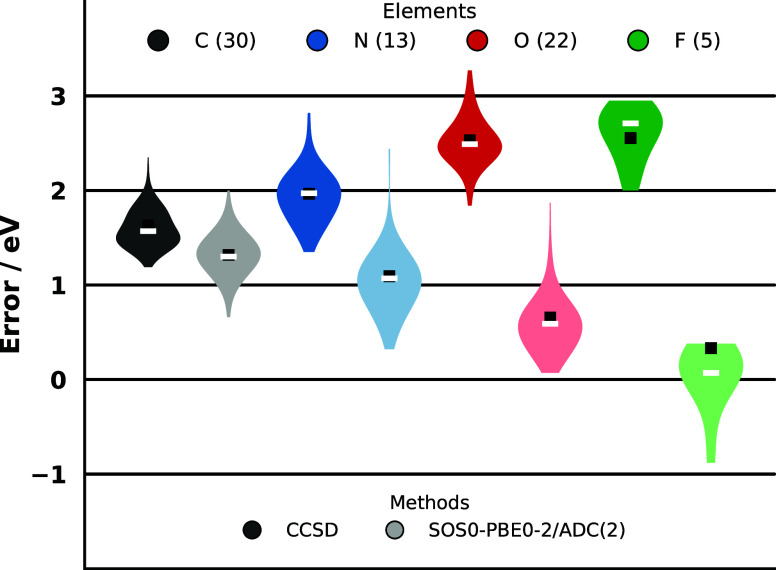
Error measures for the molecules used in this study, broken down by element. The dark (pale) color represents the results associated with the CCSD [SOS0-PBE0-2/ADC(2)] method. Further details on the technical aspects are provided in the captions of Figures 1 and 2, while information on the composition of the data set can be found in the main text. The ME and MAE are indicated by a white line and a black square, respectively.

### 4.4 Timings

From a practical standpoint, in addition to accuracy, the computational cost of the methods is of key importance. Accordingly, we present wall-clock timings for an azafullerene molecule, calculated for the N K-edge ionization following ref 58. All calculations were performed using the cc-pCVTZ-X2C basis sets. For this 61-atom molecule, this corresponds to 2594 atomic orbitals and, due to the extensive AutoAUX sets, 14,121 auxiliary functions. The results are summarized in Table 2. Examining the core binding energies, we can conclude that the present values are consistent with those obtained for smaller molecules. The ADC(2) method slightly underestimates the experimental ionization energy, whereas applying the SOS scheme introduces a serious blueshift, resulting in a pronounced overestimation for SOS-ADC(2). The DH functional incorporating the iterative second-order correction compensates for this effect by lowering the binding energy and reproduces the experimental value of 406.00 eV with an error of less than 1.7 eV. The CCSD method shows a deviation of nearly 3 eV. It is worth noting that the previously observed trend is also apparent among the CIS­(D)-based methods. The SOS-CIS­(D) approach significantly improves upon the CIS­(D) result, which is 393.62 eV, while the corresponding DH functional further refines it, bringing the value closer to experiment. Nevertheless, even this result remains about 2 eV away from the experimental value. Although this deviation is acceptable, particularly in comparison with the accuracy of CCSD, we still do not recommend using the perturbative ansatz for the reasons discussed below.

**Table 2:** N K-Edge Core Binding Energies (in eV, Upper Panel) and Wall-Clock Times (in min, Lower Panel) for Different Methods for the Azafullerene Molecule Using cc-pCVTZ-X2C Basis Sets^a^

	exp.	DMET-IP-CVS-EOM-CCSD^b^	ADC(2)	SOS-ADC(2)	SOS0-PBE0-2/ADC(2)	SOS-CIS(D)	SOS0-PBE0-2/CIS(D)
core binding energy	406.00	408.86	404.81	410.46	407.66	403.17	404.27
HF/KS reference			513.2	513.2	598.9	513.2	598.9
molecular integrals^c^			88.0	88.0	88.0	88.0	88.0
second-order calculation			1.3	1.3	1.3	0.5	0.5

a The DMET-IP-CVS-EOM-CCSD value was taken from Ref 58. All values include SFX2C-1e relativistic contributions.

b IP-CVS-EOM-CCSD combined with density matrix embedding theory.^58^

c Integral calculation and transformation to the molecular orbital basis set.

Regarding computational costs, the difference between the perturbative and iterative second-order correction schemes is negligible, and the computation of the correction itself represents only a small fraction of the total runtime. For the purely wave function-based approaches, obtaining the HF reference required slightly more than 500 min. The evaluation and transformation of the integrals necessary for the second-order calculation took approximately 90 min. It should be noted that this step is required for all electron-correlation methods and that, in our case, the virtual–virtual block of the molecular orbital integrals is not needed, which significantly reduces the time required for the transformation. In comparison, the perturbative and iterative second-order calculations themselves required only about 0.5 and 1.3 min, respectively—an insignificant fraction of the total computational time in both cases. When applying DH functionals, the determination of the KS reference orbital set takes somewhat longer, as the XC contribution must also be evaluated. However, once the KS solution is obtained, the subsequent calculations require the same amount of time as for the wave function-based counterparts since the XC terms no longer need to be computed. Based on these observations, we can conclude that once the HF/KS reference solution is available, the second-order corrections presented in this study can be computed with minimal additional computational effort.

## 5 Conclusions

In this study, the calculation of core binding energies using second-order methods has been implemented. To this end, we proposed a theoretical framework that combines the perturbative IP-CIS­(D) and iterative IP-ADC(2) approaches with the CVS approximation, thereby enabling ionization from the core region. The working equations necessary for an efficient implementation are provided. The analysis of the algorithms revealed that the approaches exhibit highly favorable scaling behavior. The computational cost of the corrections is effectively cubic, with only a mild prefactor that depends on the number of active core orbitals. The resulting CVS-IP-CIS­(D) and CVS-IP-ADC(2) methods were further extended to DH functionals. Since the XC contributions appear solely in the determination of the reference orbital set, no modifications to the implementation are required; only the second-order correction terms need to be scaled by the correlation mixing factor associated with the functional.

The performance of the proposed methods was assessed using two widely adopted benchmark sets, employing experimental ionization energies as reference data. One of these sets comprised smaller molecules, while the other was the well-known CORE65 compilation. In summary, the results demonstrate that CVS-IP-ADC(2) generally provides lower ME and MAE values than CCSD, although its precision is somewhat limited due to the larger SD and error span. The SOS-ADC(2) approach significantly improves upon this; however, the pronounced blueshift observed in the ionization energies leads to increased ME and MAE values despite the smaller SD and span. This deficiency is effectively corrected through the application of DH functionals. The SOS0-PBE0-2/ADC(2) method delivers accuracy that clearly surpasses that of the substantially more expensive CCSD approach while maintaining practically identical precision.

The study also confirms the importance of the iterative treatment of double excitations; consequently, the use of CIS­(D)-based methods is not recommended. This conclusion is further supported by the observation that the overall computational cost of the perturbative and iterative second-order methods is practically identical. Based on calculations performed for a 61-atom azafullerene molecule using cc-pCVTZ-X2C basis sets, we demonstrated that once the reference orbital set and the required molecular orbital integrals have been obtained, the evaluation of the second-order corrections takes only about one minute. Moreover, the memory requirements are exceptionally low, as the virtual–virtual block of the molecular integral list, which is necessary for higher-order methods, is not needed in our case. On top of that, the computation and storage of four-index arrays are entirely avoided within this formalism, and none of the required intermediates contains more than one virtual index.

## Supplementary Material



## Notes

Calculated core binding energies and statistical measures (XLSX)
